# The positivity of G-protein-coupled receptor-30 (GPR 30), an alternative estrogen receptor is not different between type 1 and type 2 endometrial cancer

**DOI:** 10.18632/oncotarget.18545

**Published:** 2017-06-17

**Authors:** Jiayi Wan, Yongxiang Yin, Min Zhao, Fang Shen, Miaoxin Chen, Qi Chen

**Affiliations:** ^1^ Department of Pathology, Wuxi No2 People’s Hospital, Nanjing Medical University, Wuxi, China; ^2^ Department of Pathology, Wuxi Maternity and Children Hospital, Nanjing Medical University, Wuxi, China; ^3^ The Hospital of Obstetrics and Gynaecology, Fudan University, Shanghai, China; ^4^ Centre for Reproductive Medicine, Shanghai First Maternity and Infant Hospital, Tongji University School of Medicine, Shanghai, China; ^5^ Department of Obstetrics and Gynaecology, The University of Auckland, Auckland, New Zealand

**Keywords:** endometrial cancer, GPR30, estrogen receptor, menopause, type 1 and type 2

## Abstract

It is well-known that the clinical outcomes are different between type 1 (estrogen dependent) and type 2 (estrogen independent) endometrial cancer. Studies have suggested that the estrogen receptor (ER) is positively correlated with endometrial cancer survival, however we previously reported that there is no difference in the positivity of ER as well as sex hormone levels between subtypes of cancer. G-protein-coupled receptor-30 (GPR 30), an alternative estrogen receptor has been suggested to be negatively correlated with clinical outcomes of endometrial cancer. In this study we investigated whether the positivity of GPR30 is different between subtypes of cancer. The immunostaining of GPR30 and ER was examined and analysed in 128 cases taking into account menopausal status. Overall, 105 (82%) cases were GPR30 positive and 118 (92%) cases were ER positive. The positivity of GPR30 in type 1 endometrial cancer (83%) was not statistically different to type 2 endometrial cancer (78%). In addition, intensity of immunostaining of GPR30 in type 1 endometrial cancer was also not different to type 2 endometrial cancer quantified by semi-quantitative analysis (*p* = 0.268). Menopausal status was not associated with the positivity of GPR30 in both type 1 and type 2 endometrial cancer. Furthermore, the positivity and intensity of immunostaining of GPR30 were not correlated with the positivity and intensity of immunostaining of ER in endometrial cancer (*p* = 0.689). Our data further confirm that type 2 endometrial cancer may not be completely estrogen independent, and suggest that type 1 and type 2 endometrial cancer may have similar pathogenesis.

## INTRODUCTION

Endometrial cancer has recently been a major gynaecological cancer in developed countries and causes more than 10,000 deaths in the United States yearly (American Cancer Society: Cancer Facts and Figures 2016). The exact causes of endometrial cancer are still unclear, however unopposed endometrial estrogen exposure, such as estrogen replacement therapy during menopause has been suggested to be associated with increased risk of developing this disease [1]. Endometrial cancer is traditionally divided into estrogen dependent (type 1) and estrogen independent (type 2) [2], although a new classification of endometrial cancer has recently been reported that includes four pathological subtypes of endometrial cancer based on molecular signatures [3]. Type 1 endometrial cancer is thought to be caused by excess estrogen, while type 2 endometrial cancer was not.

Estrogen and progesterone exert their effect through intra-and extra-nuclear receptors. It is well documented that the positivity of estrogen receptor (ER) and progesterone receptor (PR) is positively associated with the prognosis of endometrial cancer, including the survival rate and survival time [4, 5]. Although type 1 endometrial cancer has a better survival rate with treatment, while type 2 has a poorer prognosis with an aggressive form of the disease [2], our recent studies have found that there is no difference in the positivity of ER or PR as well as the sex hormone levels between type 1 and type 2 endometrial cancer [6, 7]. In addition, a study has hypothesised that type 2 endometrial cancer may not be completely estrogen-independent because both type 1 and type 2 endometrial cancer share many common risk factors [8]. This suggests that another factor(s) may be involved in causing the difference in clinical outcomes between type 1 and type 2 endometrial cancer.

G-protein-coupled receptor-30 (GPR 30), an alternative intra-cellular estrogen receptor was identified in 2005 and is able to mediate estrogen action [9, 10]. Unlike subunits of ER (ERα and ERβ) that function as estrogen- activated transcription factors in the nucleus and do not influence gene transcription [11], GPR30 is a transmembrane estrogen receptor which is involved in the rapid nongenomic effect of estrogen [reviewed in [12–14]] and is a specific receptor for 17β-estradiol which is a most potent estrogen subtype [12]. GPR30 is widely overexpressed in a number of cancer cells including endometrial cancer cells [15–17] and has been suggested to be a novel indicator of clinical outcomes of endometrial cancer [18]. This potentially suggests that the positivity of GPR30 may be different between type 1 and type 2 endometrial cancer.

Therefore, this study aimed to investigate the positivity of GPR30 in endometrium between type 1 and type 2 endometrial cancer taking into account menopausal status and whether the positivity of GPR30 is correlated with the positivity of ER.

## RESULTS

### Clinical characteristics of the study population

The clinical and histological characteristics of study participants are summarised in Table 1. The median age of patients at diagnosis was 56 (range 29–82) years old. Of 128 patients, 100 (78%) were diagnosed with type 1 endometrial cancer, and 44 (34%) patients were diagnosed before menopause. There was no statistical difference in the median age between premenopausal women with type 1 (55 range from 29 to 82 years) and type 2 endometrial cancer (57 range from 35 to 71 years) at diagnosis. There was also no statistical difference in the median age between postmenopausal women with type 1 (61 range from 46 to 82 years) and type 2 endometrial cancer (63 range from 47 to 71 years) at diagnosis.

**Table 1:** Clinical characteristics of the study population

	Women with endometrial cancer (*N* = 128)
Age at diagnosis (years, median/range)	56 (29-82)
Premenopause (number, %)	44 (34%)
Post- menopause (number, %)	84 (66%)
Type 1(number, %)	100 (78%)
Type 2 (number, %)	28 (22%)

### GPR30 was expressed in the luminal or basal surface of epithelium

**Figure 1:**
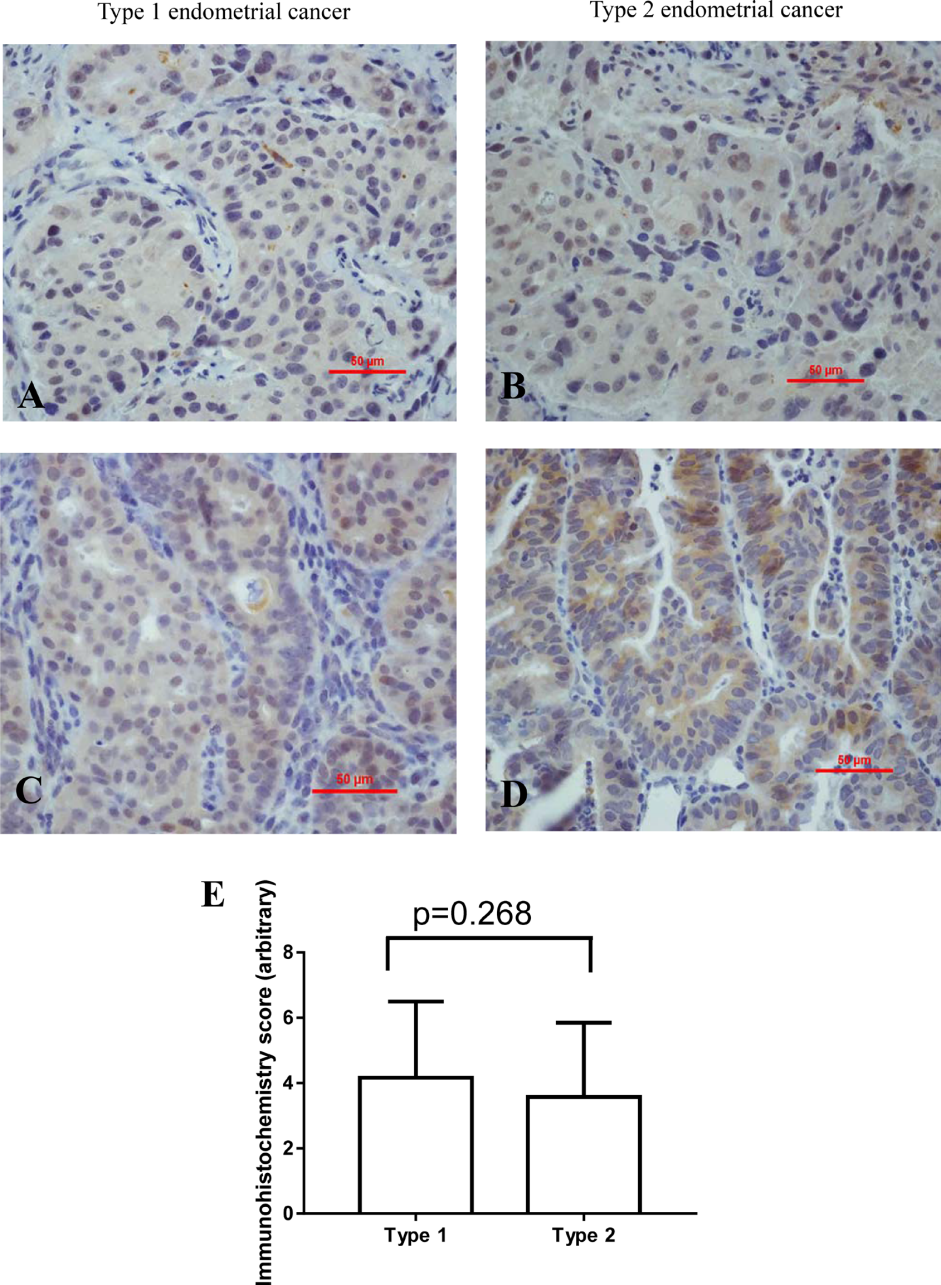
Representative immunohistochemistry images showing the immunostaining of GPR30 in type 1 (left) and type 2 (right) endometrial cancer in the luminal (**A**, **B**) or endometrial glands (**C**, **D**). Semi-quantitation of the overall immunohistochemical analysis indicated there was no difference in intensity of staining between type 1 and type 2 endometrial cancer (**E**, *p* = 0.268).

Overall, 105 (82%) cases were GPR30 positive. We then investigate the localization of GPR30. The immunostaining of GPR30 was performed by immunohistochemistry (Figure 1). GPR30 mainly expressed in the luminal (Figure 1A and 1B) or basal surface of epithelium (Figure 1C and 1D) in either type 1 or type 2 endometrial cancer. Semi-quantitative analysis of the overall immunohistochemistry staining showed that there was no difference in the intensity of immunostaining of GPR30 between type 1 and type 2 endometrial cancer (Figure 1E, *p* = 0.268).

### The positivity of GPR30 is not different between type 1 and type 2 endometrial cancer

We then analysed the positivity of GPR30 between two subtypes of endometrial cancer. In type 1 endometrial cancer, 83 (83%) cases were GPR 30 positive, whereas in type 2 endometrial cancer, 19 (78%) cases were GPR30 positive respectively (Table 2). There was no statistical difference in the positivity of GPR30 between type 1 and type 2 endometrial cancer (*p* = 0.391).

**Table 2:** The expression of estrogen receptor (ER) or progesterone receptor (PR) in endometrial cancer

	Type 1 (*n*=100)	Type 2 (*n*=28)	*P* value
GPR30 positive (number, %, lower, upper CL)	83 (83%) (75%, 90%)	22 (78%) (59%, 91%)	0.391

Menopausal status is one of the risk factors for developing endometrial cancer, we then compared the positivity of GPR30 in patients before menopause or after menopause according to the cancer types (Table 3). In premenopausal women, 32 (88.8%) cases with type 1 endometrial cancer were GPR30 positive, and 7 (87.5%) cases with type 2 endometrial cancer were GPR30 positive. There was no statistical difference in the positivity of GPR30 between type 1 and type 2 endometrial cancer in premenopausal women (*p* = 0.999). Similarly, in postmenopausal women, 51 (80%) cases with type 1 endometrial cancer were GPR30 positive and 15 (75%) cases with type 2 endometrial cancer were GPR30 positive. There was also no statistical difference in the positivity of GPR30 between type 1 and type 2 endometrial cancer in premenopausal women (*p* = 0.756).

**Table 3:** The expression of GPR30 in endometrial cancer between cancer types according to menopausal status

premenopause	Type 1 (*n* = 36)	Type 2 (*n* = 8)	*P* value
GPR30 positive (number, %)	32 (88.8%)	7 (87.5%)	*p* = 0.999
postmenopause	Type 1 (*n* = 64)	Type 2 (*n* = 20)	
GPR30 positive (number, %)	51 (80%)	15 (75%)	*P* = 0.756

### The positivity of GPR30 in endometrial cancer is not associated with menopausal status

We further investigated whether the positivity of GPR30 is associated with menopausal status taking into account cancer subtypes. As shown in Table 4, in type 1 endometrial cancer (*n* = 100), 32 (88.8%) cases were GPR30 positive in premenopausal women. 51 (80%) cases were GPR30 positive in postmenopausal women. There was no difference in the positivity of GPR30 in type 1 endometrial cancer between premenopausal and postmenopausal patients (Table 4, *p* = 0.283). In type 2 endometrial cancer (*n* = 28), 7 (87.5%) cases were GPR30 positive in premenopausal women. 15 (75%) cases were GPR30 positive in postmenopausal women. There was also no difference in the positivity of GPR30 in type 2 endometrial cancer between premenopausal and postmenopausal patients (Table 4, *p* = 0.634).

**Table 4:** The expression of GPR30 in endometrial cancer between premenopause and postmenopause according to subtypes of endometrial cancer

Type 1 (*n* = 100)	Premenopause (*n* = 36)	Postmenopause (*n* = 64)	*P* value
GPR30 positive (number, %)	32 (88.8%)	51 (80%)	*p* = 0.281
Type 2 (*n* = 28)	Premenopause (*n* = 8)	Postmenopause (*n* = 20)	
GPR30 positive (number, %)	7 (87.5%)	15 (75%)	*P* = 0.634

### The positivity of GPR30 is not correlated with ER positivity in endometrial cancer

Studies have indicated that GPR30 is an alternative estrogen receptor [9, 10]. We then investigated the correlation between the positivity of GPR30 and ER in endometrial cancer. The overall positivity of ER or GPR30 was 92% (118 of 128 cases) or 82% (105 of 128 cases), respectively. In GPR 30 negative cases (*n* = 23), there was 22 (96%) cases that were ER positive. While, in GPR30 positive cases (*n* = 105), there were 96 (91%) cases that were ER positive. There was no difference in the positivity of ER between GPR30 positive and GPR30 negative cases (*p* = 0.685, Table 5).

**Table 5:** The correlation between the positivity of GPR30 and ER in endometrial cancer

	ER positive (number, %)
GPR30 negative (*n* = 23)	22 (96%)
GPR30 positive (*n* = 105)	96 (91%)
*P* value	0.689

In addition, in the cases of GPR30 with intensity of immunostaining 1+ (*n* = 28), the percentage of ER with intensity of staining 1+, or 2+ or 3+ was 19% or 25% or 46% respectively, which was not different among the groups (Table 6). In the cases of GPR30 with intensity of immunostaining 2+ (*n* = 41), the percentage of ER with intensity of immunostaining 1+, or 2+ or 3+ was 30% or 27% or 31% respectively, which was also not different among the groups (Table 6). In the cases of GPR30 with intensity of immunostaining 3+ (n=36), the percentage of ER with intensity of immunostaining 1+, or 2+ or 3+ was 25% or 39% or 33% respectively, which was also not different among the groups (Table 6).

**Table 6:** The correlation between the expression (intensity of staining) of GPR30 and ER in endometrial cancer

		ER expression (number, %, lower, upper CL)		
		1+	2+	3+
GPR30 expression	1+ (*n* = 28)	5 (19%) (6%, 36%)	7 (25%) (10%, 44%)	13 (46%) (27%, 66%)
	2+ (*n* = 41)	12 (30%) (16%, 45%)	11 (27%) (14%, 42%)	13 (31%) (18%, 48%)
	3+ (*n* = 36)	9 (25%) (12%, 42%)	14 (39%) (23%, 56%)	12 (33%) (18%, 50%)

## DISCUSSION

GPR30, an alternative estrogen receptor has been reported to mediate the proliferative effects of estrogen in endometrial, ovarian and breast cancer cells [19]. The overexpression of GPR30 has been suggested to be negatively correlated with the clinical outcomes of endometrial cancer including survival rate and prognosis [18]. In our current study we found that the overall positivity of GPR30 in endometrial cancer was 82% in Chinese population. Another study reported that the overall positivity of GPR30 in endometrial cancer was 87% in Caucasians which was higher than Chinese population [18]. Ethnicity is one of the risk factors for developing endometrial cancer and the study suggested that Asian women with endometrial cancer have improved clinical outcomes and better survival rate compared to non-Asian women [20]. This may be associated with lower positivity of GPR30 in Chinese (Asian) population with endometrial cancer.

Endometrial cancer traditional divides type1 (estrogen dependent) and type 2 (estrogen independent) cancer. It is well-known that type 2 endometrial cancer has poorer clinical outcomes and prognosis compared to type 1 endometrial cancer, however to date whether there is a difference in the positivity of GPR30 between type 1 and type 2 endometrial cancer has not been investigated yet. In this study we interestingly found that there was no statistical difference in the positivity of GPR30 between type 1 and type 2 endometrial cancer (83% vs 78%). We further examined whether these is a difference in intensity of immunostaining of GPR30 between type 1 and type 2 endometrial cancer. However we found that there was also no difference in the intensity of immunostaining of GPR30 between type 1 and type 2 endometrial cancer. Taken together our data suggests that both positivity and intensity of immunostaining of GPR30 may not be related to the subtypes of endometrial cancer.

Menopausal status such as early menarche or late menopause is one of the risk factors for developing endometrial cancer. It is common that endometrial cancer occurs in postmenopausal women in Caucasians. However this is not the case for Chinese women. Our recent study reported that endometrial cancer also frequently (42%) occurs in Chinese women before menopause [21]. Therefore in this study we also investigated the positivity of GPR30 in women with endometrial cancer taking into account menopausal status. In our current study we found that there was no difference in the positivity of GPR30 between subtypes of cancer in both premenopausal and postmenopausal women suggesting the positivity of GPR30 was not associated with menopausal status regardless of subtypes of cancer.

As an alternative estrogen receptor, studies have suggested that the expression of GPR30 is negatively correlated with ER expression [18, 22]. We have recently reported that the overall positivity of ER in endometrial cancer was 85% in Chinese population [6], which was similar with the overall positivity of GPR30 in our current study. We also reported a significantly lower positivity of ER in type 2 endometrial cancer in postmenopausal women, which may be associated with poorer prognosis [6]. However, in this study we found that the positivity of GPR30 was not associated with menopausal status regardless of subtypes of cancer. Other studies reported that GPR30 was expressed in up to 50% of breast cancer regardless of the positivity of ER, suggesting GPR30 and ER have an independent influence on estrogen responsiveness in breast cancer [23]. This prompted us to question whether in fact there is a negative correlation between GPR30 and ER positivity in endometrial cancer. In our current study we interestingly found that the positivity of ER was not different between cases with GPR30 positive and cases with GPR30 negative. In addition, our data also showed that the intensity of immunostaining of ER was not correlated with the intensity of immunostaining of GPR30. Therefore our data suggest that the immunostaining of GPR30 was not correlated with the immunostaining of ER in endometrial cancer, similar to breast cancer.

There are however also important limitations of this study. Despite of the collection of samples over the study period in one women’s hospital with total number of 128 cases, the number of cases, in particular the number of cases with type 2 endometrial cancer is small. To increase the power, the conclusions drawn from this study would need to be further studied with large sample size. In addition, the age of menopause was self-reported and data on disease progression and survival were not available in this study.

In conclusion, to our knowledge this is the first report comparing the positivity and immunostaining of GPR30 between type 1 and type 2 endometrial cancer taking into account menopausal status. We demonstrate that the overall positivity of GPR30 was 82% and there was no difference in the positivity and intensity of immunostaining of GPR30 between subtypes of endometrial cancer. We also found the positivity of GPR30 was not associated with menopausal status as well as with the positivity of ER in endometrial cancer. Type 2 endometrial cancer is commonly described as estrogen independent which suggests that estrogenic and antiestrogenic exposures would not be related to its risk. Obesity is associated with higher levels of circulating estrogens in postmenopausal women and with lower progesterone levels in premenopausal women. However, our current study further confirms that type 2 endometrial cancer is not completely estrogen independent, and suggests that type 1 and type 2 endometrial cancer may have similar pathogenesis. Therefore risk factors that are associated with estrogen such as obesity are also important for developing type 2 endometrial cancer. In addition, using hormones or hormone-blocking drugs to treat endometrial cancer which are used to treat endometrial cancer cell with hormone receptors may also apply to type 2 endometrial cancer.

## MATERIALS AND METHODS

This study was approved by the Ethics Committee of Wuxi Maternity and Children Hospital, Nanjing Medical University of China. All patient-derived tissues were obtained with written informed consent. All methods were performed in accordance with the relevant guidelines and regulations.

### Study participants

There were in total 128 women with a primary diagnosis of endometrial cancer who consented to donate the tissue for this study from January 2010 to December 2015 from Wuxi Maternity and Children Hospital, Nanjing Medical University of China. All data including age at diagnosis, self-reported age at menopause, parity and pathological findings of endometrial cancer were collected from the electronic based medical records of patients from the hospital.

The classification of type 1 and type 2 endometrial cancer was determined by pathological examination of biopsies, including cancer histologic subtypes and grades. We classified endometrioid and adenosquamous carcinoma with grade 1 and 2 as type 1 endometrial cancer. Clear-cell, serous, mucinous carcinoma and grade 3 endometrioid carcinoma were classified as type 2 endometrial cancers, according to the classification of the International Federation of Gynaecology and Obstetrics (FIGO).

Endometrial cancer was diagnosed first by a physical examination and then endometrial biopsy. The endometrial tissue was examined histologically for characteristics of cancer including types of cancer.

### Immunohistochemistry

The immunostaining of ER and GPR30 in endometrial tissue (*n* = 128) was measured by immunohistochemistry on paraffin-embedded sections. Briefly, antigen retrieval was performed by treatment with citric acid (pH 6.0) for 20 minutes. Non-specific antibody binding was blocked by incubating with 10% fetal calf serum for 20 minutes. Mouse anti-human ER (1:100, Dako, 1D5) or rabbit anti GPR30 polyclonal antibody (1:200, Abcam, ab154069) was added for 1 hour at room temperature. Sections were then washed with phosphate-buffered saline (PBS) and incubated with biotinylated anti-mouse/rabbit IgG (Dako, Denmark) for 30 minutes, and after washing sections were then incubated with streptavidin-conjugated horseradish peroxidase (Dako, Denmark) for 30 minutes. The antigen–antibody complexes were visualised using 3,3-Diaminobenzidine (DAB) and counterstained with haematoxylin. Negative controls were incubated with the irrelevant mouse/rabbit serum.

For ER positive, the cut-off point of 1% positive cells was considered as ER positive.

### Semi-quantitative analysis of immunohistochemical staining of GPR30

15 images from each sample were taken with the microscope settings unaltered. Semi-quantitative analysis of GPR30 immunostaining in immunohistochemical images was undertaken using a previously published method based on the combination of staining intensity and the percentage of positive cells [24]. Briefly, no staining is scored as 0; 1–30% of positive cells scored as 1; 31–70% of positive cells as 2; and 71–100% of positive cells as 3. Staining intensity is rated on a scale of 0 to 3, with 0 = negative; 1 = weak; 2 = moderate, and 3 = strong. The raw data were converted by multiplying the quantity and staining intensity scores. The final score points were presented as the average. Final score 0, or 1and 2, or 3 and 4, or 6 and 9 was considered as negative, 1+, or 2+ or 3+ respectively.

### Statistical analysis

The statistical difference in positivity of ER and GPR30 in patients with type 1 or type 2 endometrial cancer in premenopausal and postmenopausal women and the correlation between ER and GPR30 positivity were assessed by the Fisher’s exact test using the Prism software package (GraphPad Software Inc, San Diego, CA, USA) with *p* < 0.05 being considered as statistically significant. Semi-quantitative analysis of immunohistochemical staining of GPR30 between type 1 and type 2 was assessed *t* test (non-parametric) using the Prism software package.
